# An Intelligent Combined Visual Navigation Brain Model/GPS/MEMS–INS/ADSFCF Method to Develop Vehicle Independent Guidance Solutions

**DOI:** 10.3390/mi12060718

**Published:** 2021-06-18

**Authors:** Heba G. Mohamed, Hatem A. Khater, Karim H. Moussa

**Affiliations:** 1 Electrical Department, College of Engineering, Princess Nourah bint Abdulrahman University, Riyadh 11671, Saudi Arabia; 2 Electrical Department, College of Engineering, Alexandria Higher Institute of Engineering and Technology, Alexandria 21421, Egypt; 3 Electrical Department, Faculty of Engineering, Horus University Egypt, New Damietta 34518, Egypt; hkhater@horus.edu.eg (H.A.K.); Khassan@horus.edu.eg (K.H.M.)

**Keywords:** smart VNBM, GPS, MEMS–INS smartphone sensors, adaptive, combined filter, data fusion

## Abstract

This paper presents an integrated navigation system that can function more efficiently than an inertial navigation system (INS), the results of which are not precise enough because of drifts caused by accelerometers. The paper’s proposed approach depends primarily on integrating micro-electrical-mechanical system (MEMS)-INS smartphone integrated sensors, the Global Positioning System (GPS), and the visual navigation brain model (VNBM) to enhance navigation in bad weather conditions. The recommended integrated navigation model, using an adaptive DFS combined filter, has been well studied and tested under severe climate conditions on reference trajectories. This integrated technique can easily detect and disable less accurate reference sources (GPS or VNBM) and activate a more accurate one. According to the results, the proposed integrated data fusion algorithm offers a reliable solution for errors in the previous strategies. Furthermore, compared to the pure MEMS–INS method, the proposed system reduces navigational errors by approximately 93.76 percent, whereas the conventional centralized Kalman filter technique reduces such errors by 82.23 percent.

## 1. Introduction

The most important issue for most vehicles is an autonomous navigation system that provides non-stop and reliable service even in the most difficult weather and physical conditions. Conventional navigation (e.g., inertial navigation system (INS), Global Positioning System (GPS), and Doppler velocity system) provide real-time positioning information, but have a high error rate during repeated positioning operations. To minimize INS navigation error, most of these conventional navigation systems use the unified Kalman filter (KF) to offer non-stop navigation [1,2,3,4,5] by using embedded INS with GPS and other multi-MEMS navigation-assisted sensors.

Although navigation system errors caused by a decrease in GPS accuracy are resolved by automated methods assisted by INS/GPS/Multi-MEMS programs, there are several other incorporated techniques to enhance such positioning systems. To reduce data positioning errors when the GPS signal quality decreases, an adaptive fuzzy networked inference system using an extended Kalman filter (EKF) is proposed [6]. Another suggested method for improving navigation accuracy relies on an integrated neuro-fuzzy (NF) method of GPS operation [7]. These methods are integrated to provide a navigation solution for short-term decreased GPS precision. An integrated INS–GPS should support the GPS when the signal encounters adverse effects such as blockages and reduce the propagation error encountered in an INS system over time. However, those incorporated techniques are not expected to function appropriately if the GPS signal is vulnerable over longer intervals due to accumulated INS error. To discover further smart integrated navigation methods, researchers have studied how animals like ants and rats can find their way home with no guided references however difficult the path [8]. The study’s findings showed that animals know the world around them and can access reliable navigation data to return home. Researchers discovered that animals have a component in their brains called the hippocampus, which stores familiar landmarks on the path they have taken [9]. When an animal approaches a location that its brain has already processed, spatial position knowledge, which is determined by the animal’s eyes, is incorporated using landmarks recorded in the animal’s brain. Visual navigation systems based on camera (eye) systems have been introduced to provide accurate navigation based on animal navigation methods [10,11,12,13]. A visual navigation method called Rat-Slam was proposed in 2004, and later the Seq-Slam method, with higher efficiency and a wider scope, was proposed. However, compared to the INS system referred to in this paper, these methods of visual navigation cannot provide accurate navigation. As mentioned previously, all of the GPS, INS, and Visual navigation systems have certain deficiencies. Therefore, this paper suggests combined filter (CF) data fusion as an integrated navigation solution. Most modern mobile devices include several micro-electromechanical system (MEMS)-INS sensors [14,15,16] with accelerometers and gyroscopes that can be used in more effective navigation strategies than INS systems as they minimize cost and scale [17]. Therefore, inspired by the animal navigation approach, this paper suggests a modern navigation system based on optimized micro-electrical-mechanical system (MEMS)-INS mobile sensors with GPS and a visual navigation brain model (VNBM). This system also constantly captures camera (eye) snapshots of the surrounding area along the road and compares them with reference points on the route to determine the exact location for position correction.

This paper can be summarized according to the following points: First, MEMS–INS mobile sensors are used as the primary navigation device to determine speed and location. Secondly, the position error of the MEMS–INS system is corrected using the GPS when the signal is available and working smoothly. Third, in the VNBM model, the moving vehicle captures snapshots that are matched to reference images on the path, using the position error of the MEMS–INS system as a reference source when the GPS signal is weak or unavailable. Finally, the paper proposes the use of the combined filter (CF) adaptive data sharing factor (DSF) to process the data fusion.

The paper is divided as follows: Section 2 describes the principles of the navigation equations of the GPS, MEMS–INS, and VNBM models. Section 3 introduces the combined filter and the ADSF principles. The explanation and argument of the sensors proposed for VNBM/GPS/MEMS–INS smartphones using the integrated technique of Adaptive DSF CF are discussed in Section 4. The experimental works and estimated outcomes are discussed in Section 5. Finally, Section 6 provides the paper’s conclusion.

## 2. Navigation Subsystem Basics and Errors Analysis

### 2.1. GPS Basics and Error Analysis

#### 2.1.1. Principles of GPS

There are 24 satellites in the GPS, positioned 20,180 km above the Earth’s surface to determine location (latitude, longitude, and height) information [18]. Any GPS device needs input from at least four satellites to determine the information regarding the correct position. A certain time of arrival from satellite to receiver can be calculated by the GPS receiver [19]. The GPS receiver position is set as follows:

(1) $$ps_{i}=r_{i}+c_{b}\;,$$

(2) g=cΔ ,

(3) $$r_{i}=\sqrt{{\left(x-x_{i}\right)}^{2}+{\left(y-y_{i}\right)}^{2}+{\left(z-z_{i}\right)}^{2}}$$

where *i* is satellite number; p is pseudo-range in meters; $\left(x_{i},y_{i},z_{i}\right)$ are receiver positions along the three axes in meters; and $c_{b}$ is the clock bias normalized by the speed of light.

#### 2.1.2. GPS Impairments

Most GPS errors are caused by multiple parameters that affect position information accuracy. Errors in GPS analysis, described in [20], are classified into six categories: satellite ephemeris, clock, ionosphere, troposphere, receiver, and multipath.

The GPS error is defined as:

(4) $$z\left(k\right)=H_{k}X\left(k\right)+u\left(k\right)$$

where *z*(*k*) is the position error; *H_k_* is the observation matrix; *X*(*k*) is the state vector; *u*(*k*) is Gaussian White Noise (GWN); and *k* is the instance time index.

### 2.2. MEMS–INS Smart-Phone Sensors and Error Analysis

#### 2.2.1. Structure of MEMS–INS

There are three gyroscopes and three accelerometers in MEMS smartphone sensors that determine the angular rates (*p*, *q*, *r*) and accelerations (a*x*, a*y*, a*z*) [21]. These parameters are used to define the attitude and velocities in the Vehicle coordinate system (VCS) frame. The attitude and velocities are denoted by (ϕ,θ,ψ),and (U,V,W), respectively. The attitude is used to determine the direction cosine matrix (DCM). The DCM converts the velocities from the VCS frame to the North-East-Up (NEU) frame [22]. The relationship between the derivative of the Euler angles (ϕ,θ,ψ) and angular rates are given by

(5) $$\left[\begin{array}{c} \dot{\phi }\\ \dot{\theta }\\ \dot{\psi } \end{array}\right]=\left[\begin{array}{ccc} 1 & \sin\phi \tan\theta & \cos\phi \tan\theta \\ 0 & \cos\phi & -\sin\phi \\ 0 & \sin\phi /\cos\theta & \cos\phi /\cos\theta \end{array}\right]\left[\begin{array}{c} p\\ q\\ r \end{array}\right]$$

The attitude (ϕ,θ,ψ) is given by integration depending upon the preliminary parameters of attitude at a certain time [23,24]. The accelerometers of MEMS smartphones serve to determine the accelerations in the VCS framework. The acceleration induced by gravity (*g*) is considered a function of the position above the Earth’s surface as highlighted in the following equation:

(6) $$\begin{array}{l} \dot{U}=a_{x}+rV-qW+g\sin\theta \\ \dot{V}=a_{y}-rU+pW-g\cos\theta \sin\phi \\ \dot{W}=a_{z}+qU-pV-g\cos\theta \cos\phi \end{array}$$

and it is integrated using the initial velocities to calculate (*U*, *V*, *W*) in the VCS frame. Then, (*U*, *V*, *W*) are converted to velocities (*V_N_*, *V_E_*, *V_U_*) from the VCS frame to the NEU frame, respectively, by using DCM. This is given as

(7) $$DCM=\left[\begin{array}{ccc} \cos\theta \cos\psi & \cos\theta \sin\psi & -\sin\theta \\ \sin\theta \sin\phi \cos\psi -\sin\psi \cos\phi & \sin\psi \sin\theta \sin\phi +\cos\psi \cos\phi & \sin\phi \cos\theta \\ \sin\theta \cos\phi \cos\psi +\sin\psi \sin\phi & \sin\phi \sin\theta \cos\phi -\cos\psi \sin\theta & \cos\phi \cos\theta \end{array}\right]$$

The relationship between these velocities is presented in

(8) $${\left[\begin{array}{c} V_{N}\\ V_{B}\\ V_{D} \end{array}\right]}_{INS}=DCM^{T}{\left[\begin{array}{c} U\\ V\\ W \end{array}\right]}_{INS}$$

**Figure 1:**
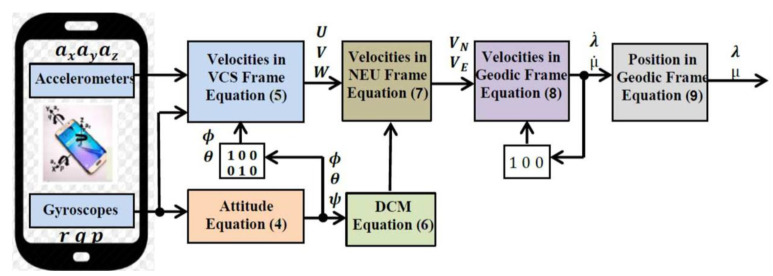
Algorithm of MEMS–INS navigation.

In this article, the geodetic (latitude, longitude, altitude) frame is used as a navigation frame. Let λ,μ, and  ħ indicate the latitude, longitude, and altitude of the vehicle, respectively. The relationship between the geodetic frame and velocities (*V_N_*, *V_E_*, *V_U_*) in the NEU frame is given in

(9) $${\left[\begin{array}{c} \dot{\lambda }\\ \dot{\mu }\\ \dot{ћ} \end{array}\right]}_{INS}=\left[\begin{array}{ccc} \frac{1}{R_{e}} & 0 & 0\\ 0 & \frac{1}{R_{e}\cos\lambda } & 0\\ 0 & 0 & -1 \end{array}\right]{\left[\begin{array}{c} V_{N}\\ V_{E}\\ V_{U} \end{array}\right]}_{MEMS}$$

where *Re* is the Earth’s radius. By integrating and using the preliminary parameters of a location at a certain time, (9) gives the location λ,μ, and  ħ in the geodetic frame. The algorithm of MEMS–INS smartphone navigation is shown in Figure 1.

#### 2.2.2. MEMS–INS Analysis Errors

In the MEMS–INS system, incorrect arrangement angles, velocity, and location errors are represented as state variables that are identified in

(10) $$\dot{X}=A_{0}X$$

where the included elements are defined by

(11) $$X={\left[\begin{array}{c} \delta P_{G}^{T}{}_{\;}\;\;\;\;\delta P_{B}^{T}\;\;\;\;\phi^{T}\;\;\;\;\nabla^{T}\;\;\;\;\varepsilon^{T} \end{array}\right]}^{T}$$

(12) $$A_{0}=\left[\begin{array}{ccccc} A_{P_{G}P_{G}} & A_{P_{G}P_{B}} & O_{3\times 3} & O_{3\times 3} & O_{3\times 3}\\ A_{P_{B}P_{G}} & A_{P_{B}P_{B}} & A_{P_{B}} & A_{P_{B}\nabla } & O_{3\times 3}\\ A_{\phi P_{G}} & A_{\phi P_{B}} & A_{\phi \phi } & O_{3\times 3} & A_{\phi \varepsilon }\\ O_{2\times 2} & O_{2\times 2} & O_{3\times 3} & O_{3\times 3} & O_{3\times 3}\\ O_{2\times 2} & O_{2\times 2} & O_{3\times 3} & O_{3\times 3} & O_{3\times 3} \end{array}\right]$$

where $\delta P_{G}={\left[\delta \lambda_{G}\;\delta \mu_{G}\right]}^{T}$signifies the error of latitude and longitude coordinates between the two systems, MEMS–INS and the GPS. $\delta P_{B}={\left[\begin{array}{c} \delta \lambda_{B}\;\delta \mu_{B} \end{array}\right]}^{T}$denotes the same error between MEMS–INS and VNBM. The $\Phi ={\left[\phi \;\theta \;\psi \right]}^{T}$denotes the attitude (roll, pitch, yaw) incorrect arrangement angles, in turn. The $\nabla ={\left[\nabla_{x}\;\nabla_{y}\;\nabla_{z}\right]}^{T}$denotes accelerometer bias and $\varepsilon =\left[\varepsilon_{x}\varepsilon_{y}\varepsilon_{z}\right]$ denotes the gyro drift. The $O_{3\times 3}$ indicates a 3 × 3 matrix with zero value; $A_{ij}\left(i=P_{G},P_{B},\phi ,j=P_{G},P_{B},\phi ,\nabla ,\varepsilon \right)$ signifies the transform matrix *j* and *i*. The following set of equations from (13) to (22) are used in the conversion process:

(13) $$A_{P_{G}P_{G}}=\left[\begin{array}{cc} 0 & 0\\ \lambda_{G}\sec\mu \tan\mu_{G}/R & 0 \end{array}\right]$$

(14) $$A_{P_{G}P_{B}}=\left[\begin{array}{ccc} 0 & 1/R & 0\\ \sec\mu \tan\lambda_{G}/R & 0 & 0 \end{array}\right]$$

(15) $$A_{P_{B}P_{G}}=\left[\begin{array}{cc} 2w_{iey}^{n}\lambda_{B}+\lambda_{B}\mu_{B}\sec^{2}\mu_{B}/R & 0\\ -\left(2w_{iey}^{n}\mu_{B}+\mu_{B}^{2}\sec^{2}\mu_{B}/R\right) & 0 \end{array}\right]$$

(16) $$A_{P_{B}P_{B}}=\left[\begin{array}{cc} \lambda_{B}\tan\mu_{B}/R & 2w_{iez}^{n}+V_{E}\tan\mu_{B}/R\\ -\left(2w_{iez}^{n}+\lambda_{R}\tan\mu_{B}/R\right) & 0 \end{array}\right]$$

(17) $$A_{\phi P_{G}}=\left[\begin{array}{cc} 0 & 0\\ -w_{ieZ}^{n} & 0\\ w_{iey}^{n}+V_{E}\sec^{2}\lambda_{G}/R & 0 \end{array}\right]$$

(18) $$A_{\phi P_{B}}=\left[\begin{array}{cc} 0 & -1/R\\ 1/R & 0\\ \tan\mu_{B}/R & 0 \end{array}\right]$$

(19) $$A_{\phi \phi }=\left[\begin{array}{ccc} 0 & w_{iez}^{n}+V_{E}\tan\mu_{G}/R & 0\\ -\left(w_{iez}^{n}+V_{E}\tan\mu_{G}/R\right) & 0 & -\frac{\lambda_{G}}{R}\\ w_{iey}^{n}+\frac{\mu_{G}}{R} & \frac{\lambda_{G}}{R} & 0 \end{array}\right]$$

(20) $$A_{V\phi }=\left[f^{n}X\right]$$

(21) $$A_{V\nabla }=DCM_{VCS}^{NEU}$$

(22) $$A_{\varepsilon \phi }=DCM_{VCS}^{NEU}$$

where $R=R_{m}$ is the radius of the Earth and $DCM_{VCS}^{NEU}$ changes the direct convert matrix from the vehicle NEU frame to the NCS frame. The $w_{iey}^{n}=\cos\mu_{G}\;\mathrm{and}\;w_{iez}^{n}=w_{ie}\sin\mu_{G}$ represents the angular velocities of the Earth’s rotation along the *oy* and *oz* axes, respectively.

### 2.3. VNBM and Error Analysis

#### 2.3.1. Principle of VNBM

**Figure 2:**
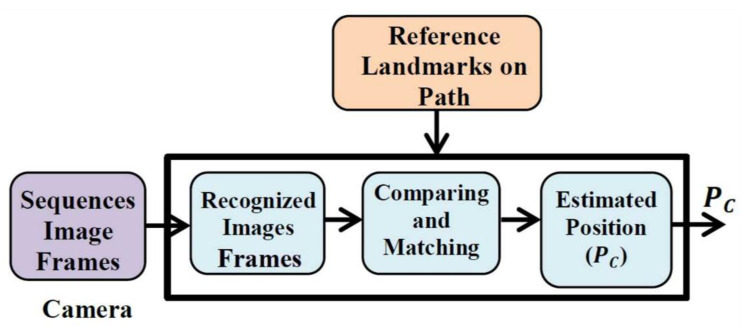
Block Diagram of the VNBM System.

The VNBM block diagram is depicted in Figure 2. The camera in this system is based on animal eyes that capture pathway images from the surrounding environment and relies on path reference landmarks that are similar to the stored animal brain navigation data.

The VNBM system is arranged as follows: First, the camera captures sequence images from the surrounding environment on the path [25,26]. Second, the captured images are compared and matched with reference landmark images [27]. Finally, when the matching process shows them to correspond, the estimated position can be calculated using the coarse-to-fine (CTF) method [28] in which the image frames are matched in temporally consecutive sequences. The localized coarse is provided by reducing the region (T) to obtain the coarse place field (H) that gives the best matching image places.

(23) $$T=T\left(p\left(k\right)+\Delta p^{*}\right)$$

where p(k) and $p^{*}$ are the current time position and the predefined region, respectively. The image place matching at the smallest scale sc in the coarse region is given by

(24) [H,H+sc−1]

The best matching result for the coarse matching region is *n* with different scores$\;D\left(Y_{n}\right)$. The lowest score is determined as the final matching result. Based on the CTF method, each placed image is marked with the corresponding position. Therefore, from the lowest image score, the estimated position $P_{C}$ of the examined images is given by

(25) $$P_{C}=p\left(\begin{array}{c} (\arg minD\left(Y_{n}\right))\\ n \end{array}\right)$$

#### 2.3.2. Error Analysis for VNBM

**Figure 3:**
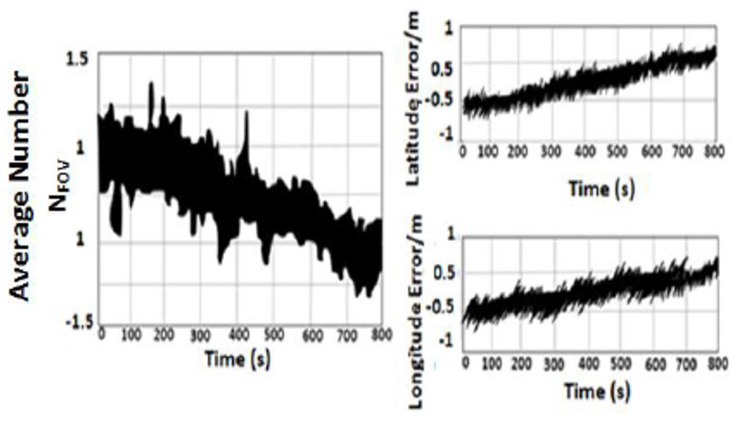
Experimental test of VNBM Accuracy.

The accuracy of VNBM is much better compared to that of the MEMS–INS smartphone. Therefore, VBNM can correct the position error of MEMS–INS. However, the field of view (FOV) of the camera and the weather conditions, such as light intensity and fog, are very important factors that affect the accuracy of the VNBM model. The FOV of the camera is given by

(26) $$N_{FOV}=6.57e^{1.08}\frac{1-\cos\left(A/2\right)}{2}$$

where $N_{FOV}$ is the average number of captured images on the path and A is the dimension of the captured image. The Field of View is an angle that depends on the focal length and sensor size, but it also computes the dimensional field of view sizes (width, height, or diagonal) at a specific subject or background distance. The 300 mm lens with matching 35 mm film has an equivalent focal length of 300 mm. Therefore, the VNBM accuracy decreases as the average number of the captured images is reduced, for example, by weather conditions such as light intensity and fog. Therefore, to determine the accuracy of VNBM, an investigational test was implemented. In the experiment, the highly accurate GPS and VNBM model were installed on a vehicle that navigated along a reference trajectory for about 800 s. The estimated location errors of the VNBM model are shown in Figure 3. During the experimental test, weather factors changed the position accuracy of VBNM. The location error depended on the average number of captured images in FOV.

The relationship between the average of NFOV and the VNBM location error is considered by fitting information (using Matlab), and it is given by

(27) $$y\left(t\right)=0.0636x^{3}+1.4732x^{2}-78.2011x+632.6667$$

## 3. Combined Filter Based on Adaptive Data Sharing Factor (ADSF)

### 3.1. Principle of Combined Filter (CF)

**Figure 4:**
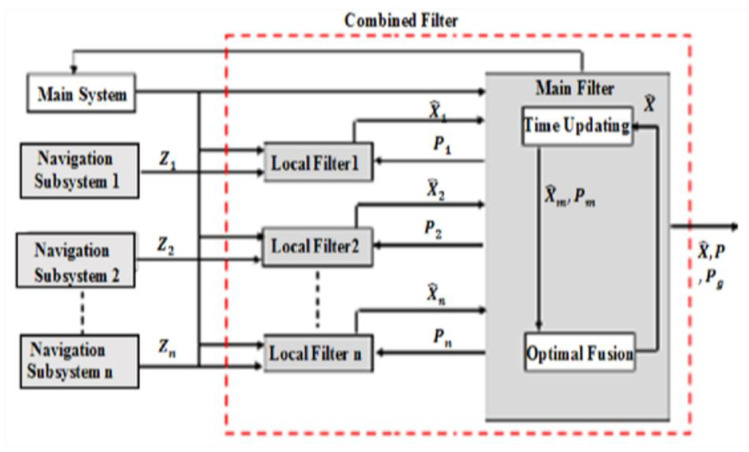
Combined filter basics.

Multi-sensor navigation system data are fused using two popular integrated methods: centralized Kalman filter (KF) and combined filter (CF). The MEMS–INS position errors detected by centralized KF, as observed variables, are corrected by the GPS and VNBM information, assuming that the GPS and VNBM are more accurate than the MEMS–INS smartphone system. The MEMS–INS navigation error resulting from (10) is, thus, expected to improve the MEMS–INS system accuracy. The combined filter (CF) is widely used because of its flexible design and good real-time performance [29,30,31,32,33,34,35,36,37]. In general, the structure of CF depends on a double-stage data processing technique. In stage one, the local Kalman filters (KF) are linked to specify the position information through navigation subsystems. In stage two, the key filter processes are blended and merged locally with the Kalman filters (KF). Figure 4 shows the integrated CF method.

Every local Kalman filter was related to one of the navigation subsystems in Figure 4. Furthermore, in each subsystem, the central filter checked the knowledge errors [38]. When one of them has its accuracy decreased, this subsystem’s mean squared error matrix was modified and increased. According to the key combined filter equations, the navigation subsystem’s error calculation, inferred by a feedback flow, was modified by global covariance. Using local Kalman filter 1 as an example here, local KF1 covariance changed as in

(28) $$\begin{array}{c} P_{1}^{-1}=B_{1}[P_{1K}^{-1}{\left(I-K_{1}H_{1}\right)}^{-1}+P_{2K}^{-1}{\left(I-K_{2}H_{2}\right)}^{-1}+\\ \ldots \ldots P_{nK}^{-1}{\left(I-K_{n}H_{n}\right)}^{-1}] \end{array}$$

when $P_{1}^{-1}$ is calculated separately. It is signified as

(29) $$P_{1}^{-1}=P_{1K}^{-1}{\left(I-K_{1}H_{1}\right)}^{-1}$$

Calculating the local covariance *P*1 by global output is shown as

(30) $$\begin{array}{c} P_{1}^{-1}=[P_{1K}^{-1}{\left(I-K_{1}H_{1}\right)}^{-1}+P_{2K}^{-1}{\left(I-K_{2}H_{2}\right)}^{-1}+\\ \cdots \cdots P_{nK}^{-1}{\left(I-K_{n}H_{n}\right)}^{-1}]{\left(I-K_{1}H_{1}\right)}^{-1} \end{array}$$

If $P_{1}^{-1}$ is independently considered, it will only be influenced by local filter 1. This implies that when the precision of a single navigation subsystem declines, the $P_{1}^{-1}\;$cannot be updated via the key combined filter, and the local filter 1 error cannot be updated. However, the $P_{1}^{-1}\;$can be refined by the main combined filter via the steady DSF calculated by the global output. Although the CF-based integrated approach may be resolved by key filter errors in any navigation device, the CF’s constant DSF cannot tailor its importance to the specific navigation error that will impact the overall navigation system’s performance. Therefore, the proposed system introduces the adaptive data sharing factor (ADSF) as a new contributing parameter.

These terms are primarily determined from the merged filter. The central unified filter reflects the ratio value of every navigation subsystem (GPS and VNBM), which implies that the ratio value of navigation subsystem I will rise in the central combined filter stage and fall when it decreases. It also implies that the value of navigation subsystem I and all other navigation subsystems with greater precision should be set to a greater ratio value in the key integrated filter stage. Therefore, the influence of the lower-accuracy navigation subsystem rendered the integrated navigation device more reliable. Based on the aforementioned method, the proposed adaptive DSF combined filter (CF) was avoided and separated by the lower navigation subsystem. The one with the best accuracy used an integrated navigation reference information method. Integrated VNBM/GPS/MEMS–INS focusing on the adaptive DSF combined filter as a data fusion approach is, therefore, the best choice for providing a safe and efficient navigation framework. In line with the principle of our proposed ADSF combined filter, the adaptive parameter was made to separate and avoid navigation subsystems with lower accuracy, which ultimately improved the performance of the entire navigation system.

### 3.2. Adaptive Data Sharing Factor (ADSF) of Combined Filter (CF)

The core parameter of the combined filter is this research’s main contribution because managing the precision of VNBM and DVL is the main problem influencing the precision of the overall navigation device. Research, therefore, suggestd that the adaptive DSF can manage the accuracy of the GPS and VNBM. This requires an expansion of the adaptive DSF. The ADSF values are set according to

(31) $$C_{ik}=H_{ik}P_{ik}^{-}H_{ik}^{T}+R_{ik}^{-}$$

where $C_{ik}$, $R_{ik}^{-}$, and $H_{ik}^{T}$ are the Kalman filter’s co-variance of invention, calculation co-variance, and calculation matrix, respectively. As seen in Figure 4, *i* = 1, 2, ... *n* means local KF1, KF2, and KFn.

Miscalculation errors, such as uncounted fault prejudice and unknown condition variables, resulted in the creation of a CF that was subject to their effect as they are directly included in the breakthrough equations. For example, if the right dynamic equations are identified, the invention’s co-variance is equal. Since unknown data have a similar effect when the precise equations of a calculation are not available, the $C_{ik}$ will increase. The change in $C_{ik}\;$can be used in the adaptive filter, and the increased innovation co-variance ${\bar{C}}_{ik}$ is given as

(32) $$\bar{C}ik=\frac{1}{M-1}\sum_{j=K-M+1}^{K}\eta_{ij}I_{ij}^{T}$$

where *M* is the size of the window that refers to the sampling frequency and performance of every navigation subsystem. The relation between the $C_{ik}\;$and ${\bar{C}}_{ik}\;$is illustrated in the following equation:

(33) $$\alpha_{ik}=\left|\mathrm{tr}\left(C_{ik}\right)-\mathrm{tr}\left(C_{ik}\right)\right|$$

where tr( ) indicates the trace of the matrix. This is the mathematical relationship between the two covariances that provides the constancy of the predestined result. The value can be verified when the precise measurement noise is known and approximated to zero. Nevertheless, while the noise modification measurement occurred abruptly and the discovered accuracy decreased at time factor *k*, the time point *k* will differ from the remaining period and the value will rise. The ADSF is therefore configured as in

(34) $$B_{ik}=\frac{\alpha_{ik}^{{}^{-1}}}{\sum_{i=1}^{2}\alpha_{ik}^{{}^{-1}}}$$

This implies that whenever a significant gap is found between the setting value and the noise calculation, the result is poor and the contribution of this local filter reduces the main integrated filter. Likewise, where there may be a moderate disparity between the set-value and the calculation noise, the result is shown to be successful, and the efficacy of this local filter is far better than that of the primary filter. The adaptive DSF is, consequently, an excellent adaptive parameter for the combined filter that could enhance the steadiness and accuracy of the combined filter as a whole = 0.

## 4. Proposed Multi-MEMS Integrated Navigation Method Using the Adaptive DSF Combined Filter

**Figure 5:**
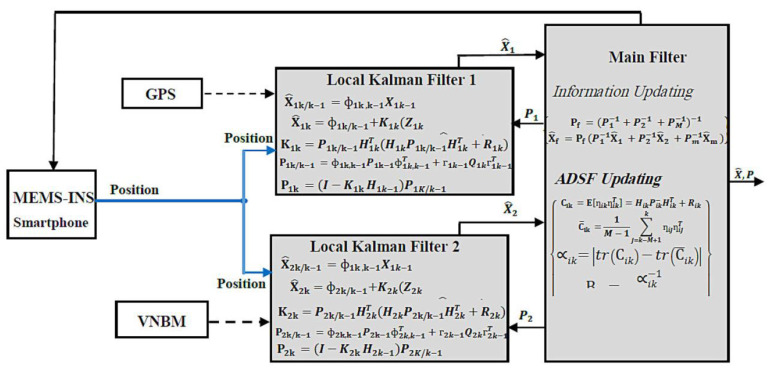
Proposed VNBM/GPS/MEMS–INS integrated method based on adaptive DSF combined filter data fusion.

The design of the integrated VNBM/GPS/MEMS–INS data fusion-dependent navigation system adaptive DSF hybrid filter is presented in Figure 5. The MEMS–INS smartphone is the key system used by the suggested integrated technique. The GPS and the VNBM serve as reference navigation subsystems to rectify the MEMS–INS location errors. Our suggested optimized approach offers the following dynamics and estimated equations:

(35) $$\dot{X}\left(t\right)=A\left(t\right)X\left(t\right)+w\left(t\right)$$

(36) $$Z_{i}\left(t\right)=H_{i}\left(t\right)X\left(t\right)+v_{i}\left(t\right)$$

where w(t), X(t) and $v_{i}\left(t\right)$ denote the estimated error, condition variable, and condition error matrices, respectively. *A*, *H,* and *Z* symbolize the state transition matrix, the measurement matrix, and the measurement equation of the local KF, in turn. The variable state is presented by

(37) $$X={\left[\delta P_{G}^{T}\;\delta P_{B}^{T}\;\phi^{T}\;\nabla^{T}\;\varepsilon^{T}\right]}^{T}$$

where $\delta P_{G}={\left[\delta \lambda_{G}\;\delta \mu_{G}\right]}^{T}$represents the latitude and longitude error between the MEMS–INS and GPS; $\delta P_{B}={\left[\delta \lambda_{B}\;\delta \mu_{B}\right]}^{T}$ denotes the latitude and longitude coordinate error between the MEMS–INS and VNBM. The state transition matrix A is given by

(38) $$A=\left[A_{0\left(13\times 13\right)}O_{13\times 2}\right]$$

From (36) *i* = 1 and 2, and represents the number of Kalman filters. The measurement matrixes *H*1 and *H*2 of both local Kalman filters are given by:

(39) $$H_{1}=\left[I_{2\times 2}O_{2\times 13}\right]$$

(40) $$H_{2}=\left[\begin{array}{c} O_{2\times 2}\;\;\;\;I_{2\times 2}\;\;\;\;O_{2\times 9}\;\;\;\;-I_{2\times 2} \end{array}\right]$$

The observation of the measurement equations of the local KF1 describing the place difference between the MEMS–INS and the GPS is given by:

(41) $$Z_{1}=\left[\lambda_{MEMS}-\lambda_{G}\mu_{MEMS}-\mu_{G}\right]$$

Similarly, the inferences of the measurement equations of local KF2, which highlights the difference in velocity between the MEMS–INS and the VNBM, is presented by

(42) $$Z_{1}=\left[\lambda_{MEMS}-\lambda_{B}\mu_{MEMS}-\mu_{B}\right]$$

Based on Figure 5, the observed local KF1 parameter signified the distinction in location between the MEMS–INS smartphone and the GPS, while the observed local KF2 parameter represented the distinction in velocity between the MEMS–INS smartphone and the VNBM. The two local Kalman filters used the calculated effects as key filter data input to approximate the final output. Next, the final approximate result was fed back into the optimized central filter to fix the MEMS–INS mistake. At the same time, the adaptive DSF adjusted local KF1 and local KF2, respectively, using the input data of P1 and P2. Assuming that GPS precision was reduced at time point k in certain pockets on the Earth’s surface, then the R1K value was inexact. Therefore, the P1, representing the approximate accuracy of the local KF1, rose to allow the C1k to increase at time factor k relative to the average from time factor k-M up to time factor k. This triggered a decrease in B1 and the input to the local KF1. Consequently, the local KF1 P1 is effective, and the main filter data input was also updated. Similarly, since the VNBM‘s accuracy fell at time factor k due to poor weather conditions, it implied that the R2K value was inexact. Then, P2, representing the approximate accuracy of local KF2, increased, allowing the C2k to increase at time factor k relative to the average of time factor k-M up to time factor k. This eventually triggers a decrease in B2 and input data to local KF2. Lastly, the local KF2 P2 proved to be efficient, and the main filter data input was also updated. While adaptive DSF combined filter processing data is more complicated than the centralized Kalman filter (KF) and constant DSF-combined filter (CF), any decrease in the GPS or VNBM precision would be sensed and differentiated by the adaptive DSF. Likewise, the easy-to-work navigation subsystems that have the highest precision are used as reference sources to enhance the navigation system. The necessary prerequisite for obtaining a highly stable and accurate navigation device solution based on VNBM/GPS/MEMS–INS is then accomplished using an adaptive DSF parameter input in the integrated data fusion of the combined filter.

## 5. Experimental Work and Results

### 5.1. Integrated Navigation Methods

**Figure 6:**
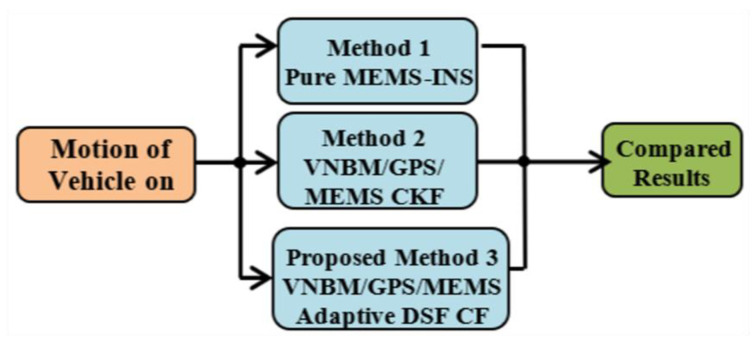
Structure of the three integrated methods.

Three integrated navigation techniques were used to compare their consequent results as presented in Figure 6. The three techniques were MEMS–INS, VNBM/GPS/MEMS–INS using a centralized Kalman filter (KF) integrated system, and the suggested integrated VNBM/GPS/MEMS–INS process using the adaptive DSF combined filter. Compared to the other two integrated techniques, the suggested technique had a highly stable and consistent navigation system resolution regarding the estimated results when the accuracy of the GPS or VNBM was reduced.

### 5.2. Hardware and Reference Trajectory

**Figure 7:**
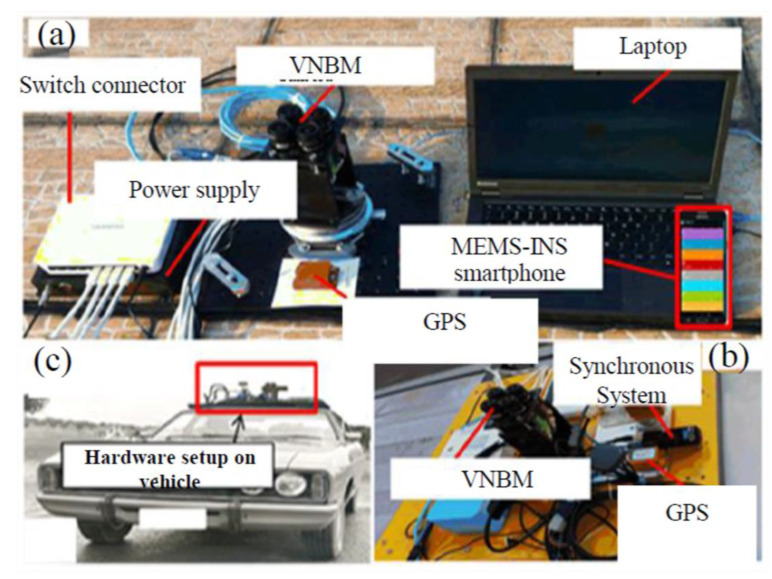
Description of hardware used: (**a**) hardware components, (**b**) hardware connection system, (**c**) hardware setup on vehicle.

**Figure 8:**
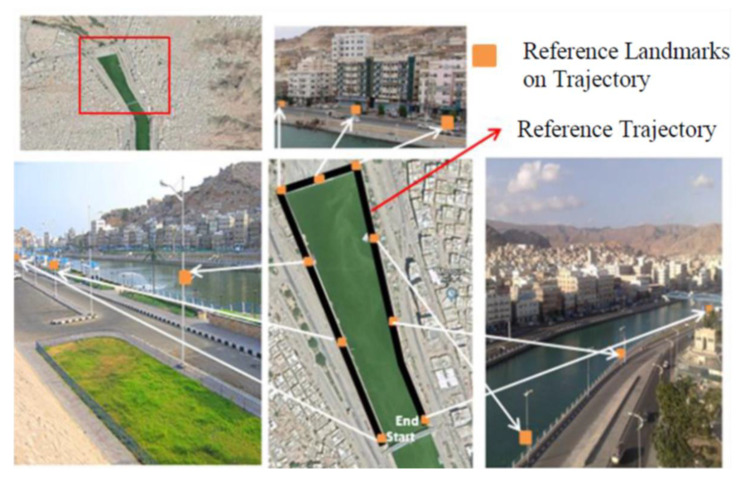
Reference trajectory and reference landmarks.

The mobile GPS, MEMS–INS (accelerometers and gyroscopes), VNBM, and other coordinated components are mounted onto the vehicle as shown in Figure 7. To prove the efficiency of the proposed method, the three integrated methods were tested under bad weather conditions, for approximately 800 s. A total of 9 reference landmarks were placed on the 800 m reference trajectory, as seen in Figure 8, which corresponded to the retained navigation data in the animal brain.

### 5.3. Parameter Setting of Three Integrated Methods

Three integrated methods were included in the study. In Method 1, pure MEMS–INS was introduced as a navigation technique without any integrated system as illustrated in Section 2. In Method 2, the VNBM/GPS/MEMS–INS centralized KF technique was presented as an integrated navigation system. The measurement matrix and dynamic model of Method 1 is given by

(43) $$H=\left[\begin{array}{c} I_{2\times 2}\;\;\;\;O_{2\times 2}\;\;\;\;O_{2\times 9}\;\;\;\;O_{2\times 2}\\ O_{2\times 2}\;\;\;\;I_{2\times 2}\;\;\;\;O_{2\times 9}\;\;\;\;-I_{2\times 2} \end{array}\right]$$

The observed calculation of (44) reflects the position error between the MEMS–INS and GPS, and the position error between the MEMS–INS and VNBM. It is illustrated as

(44) $$Z={\left[\begin{array}{cc} \lambda_{MEMS}-\lambda_{G} & \mu_{MEMS}-\mu_{G}\\ \lambda_{MEMS}-\lambda_{B} & \mu_{MEMS}-\mu_{B} \end{array}\right]}^{T}$$

In this step, the primary value of the covariance matrix had to be set before navigation could be processed. This represented the MEMS–INS smartphone error that was provided by

(45) $$Q_{0}=\mathrm{diag}\left({\left[3\times 10^{-5}3\times 10^{-5}6.73\times 10^{-7}6.73\times 10^{-7}6.73\times 10^{-7}\right]}^{2}\right)$$

In this method, the primary value of the mean square error matrix ($P_{0}$) had to be stable enough for its consistency to be tested according to the Kalman filter features. This was provided by

(46) $$\begin{array}{c} P_{0}=\\ \mathrm{diag}([0.20.21.75\times 10^{-6}1.75\times 10^{-6}1.85\times 10^{-4}1.85\times 10^{-4}\\ 1.85\times 10^{-4}3.76\times 10^{-8}3.76\times 10^{-8}3.76\times 10^{-8}\\ {1.30\times 10^{-4}1.30\times 10^{-4}1.30\times 10^{-4}0.20.2]}^{2}) \end{array}$$

For this approach, the initial values of the covariance calculation noise matrix were around 0.5 and 0.7 for the magnitude of Gaussian white noises for the GPS and VNBM measurement meters, respectively. This is provided by

(47) $$R_{0}=\left({\left[\begin{array}{c} 1/Re\;\;\;\;1/Re\;\;\;\;0.5\;\;\;\;0.7 \end{array}\right]}^{2}\right)$$

The last was Method 3, in which the VNBM/DVL/MEMS–INS adaptive DSFCF was represented as an integrated navigation system. The preliminary values of the CF could examine the setting value in Method 2. The difference between the first and the second methods was illustrated in this integrated technique. However, in Method 3 the integrated method depended on the adaptive DSFCF discussed in Section 3.

### 5.4. MEMS–INS, VNBM, and DVL Errors

The noise of the gyroscope is Gaussian white noise (GWN) with an amplitude of about 0.005° per hour, while the gyroscope drift is about 0.05° per hour. Similarly, the noise of the accelerometer GPS and VNBM are also GWN. The GPS and VNBM operated smoothly from time 0 to 310 s. Conversely, the precision of the GPS and VNBM systems was affected in the period from 310 to 620 s along the reference trajectory due to bad weather. The specifications for the navigation subsystem are listed in Table 1.

### 5.5. Comparison Results of USV Navigation Systems

**Figure 9:**
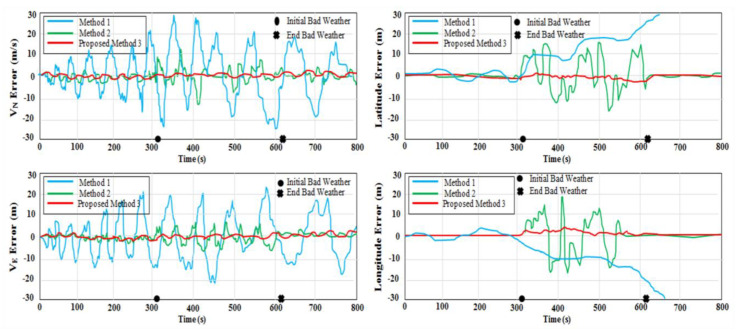
Estimated Errors with the Three Integrated Methods.

**Figure 10:**
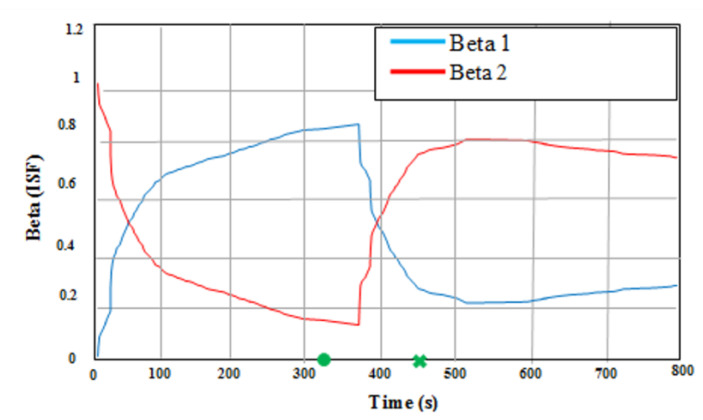
Reference Trajectory and Reference Landmarks.

The aforementioned results of the three integrated techniques are shown in Figure 9. In Method 1, pure MEMS–INS was introduced as a navigation technique without any integrated system. In Method 2, the VNBM/GPS/MEMS–INS centralized KF technique was presented as an integrated navigation system. The preliminary values of the CF could examine the setting value in Method 2. The difference between the first and the second methods is illustrated in this integrated technique. The last method was Method 3, in which the VNBM/DVL/MEMS–INS adaptive DSFCF was represented as an integrated navigation system, which depended on adaptive DSFCF. The precision of the GPS and VNBM was influenced by the duration of 310 s labeled as (●) up to 620 s labeled as (X) due to bad weather along the reference trajectory. Figure 10 shows the adaptive data sharing factor (ADSF) values. According to Figure 9, during bad weather, the root mean square error (RMSE) of the position (106.75 m) in the first method increased over time from accelerometer drifting. In the second method, the RMSE position error (15.65 m) was less than the position errors of the first method. This is because the position errors of MEMS–INS were amended by the VNBM/GPS-centralized Kalman filter. Nevertheless, this predicted result was not accurate enough during bad weather because the centralized KF technique could not precisely detect and differentiate the less accurate reference source from the more accurate one to rectify the MEMS–INS system position error. This affected update processing of the main combined filter and the general navigation system as well. In Method 3 (the proposed method), the estimated trajectory was nearly identical to the reference trajectory, and its RMSE position errors (1.53 m) were low compared to those of Method 1 and Method 2 because the adaptive DSF was precisely adjusting its values in relation to the specific errors of the navigation subsystem. Consequently, the main combined filter could be used to update the necessary values, as it accurately specified the navigation error.

In this technique, whenever the precision of the GPS declined, due to bad weather, the assessed accuracy of the local KF1, represented by the P1, increased. The B1 will then be decreased and, accordingly, the consequent data. Therefore, the P1 of the local KF1 was modified and the entered data of the main filter was updated too. Similarly, when the precision of VNBM decreased, P2, which represented the estimated accuracy of the local KF2, rose. B2 was then decreased causing the feedback data to decrease. Finally, the P2 of local KF2 was corrected and the input records of the main filter updated. Similarly, when the accuracy of VNBM decreased, P2, which represented the expected precision of local KF2, increased. B2 was suppressed, making the feedback data decrease. Finally, the P2 of thelocal KF2 was corrected and the input data of the main filter updated. In line with the adaptive DSF values in Figure 10, if the precision of the GPS were greater than that of the VNBM, Beta 1, representing the ratio of the accuracy of the GPS in the main combined filter, would rise; accordingly, Beta 2, which signifies the ratio of the precision of the VNBM, would decrease. Similarly, if the ratio of the precision of the VNBM were higher than that of the GPS, Beta 2, signifying the ratio of the precision of the VNBM in the main combined filter, would rise and, simultaneously, Beta 1, which represented a decrease in the precision level of the GPS, would decrease.

**Figure 11:**
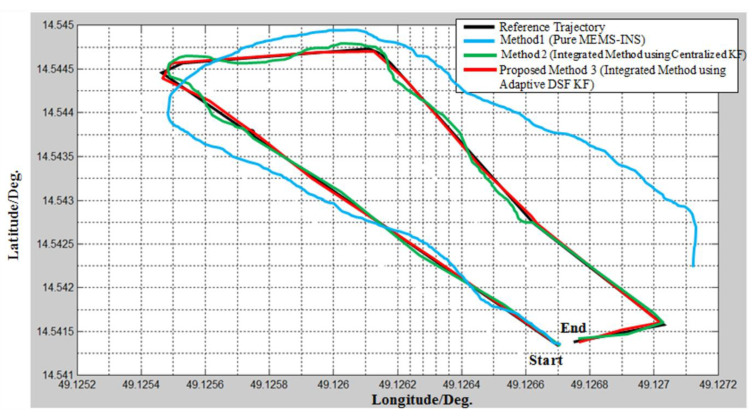
Estimated trajectories with the three integrated methods.

This proves that more reliable navigation subsystems required a greater area when upgrading the database. Thus, the less accurate navigation subsystems could be isolated and removed while modifying the main combined filter. The total approximate location (latitude and longitude) errors of the three techniques caused by bad weather from 310 to 620 s are listed in Table 2. The approximate trajectories of the three handled approaches are shown in Figure 11.

## 6. Conclusions

The proposed integrated method can provide accurate navigation solutions in urban areas and in adverse weather conditions when the GPS signal is weak or inaccessible. The system is based on the visual navigation brain model (VNBM), which relies on images captured by the camera (eyes) from the surrounding environment along a path and the subsequent matching with reference landmarks on the reference trajectory. Therefore, the system provided an accurate position to rectify the position error of the integrated navigation system and was based on a novel integrated method using adaptive data-sharing factor (ADSF) combined filter (CF) data fusion. Based on the approximate results in Table 2, the expected error in the location of the proposed integrated system was very low relative to the two other integrated approaches. The error was reduced by 95.76% compared to Method 1 and by 82.23% compared to Method 2. Furthermore, the approximate location error of ADSFCF was restored in the MEMS–INS system to reduce its error and increase the precision of the overall navigation device. Further experiments to apply the proposed system in severe weather conditions are highly recommended.

## Figures and Tables

**Table 1:** Specifications of the navigation subsystems.

Parameter	Index
Gyroscopes Dynamic Range	±100°/s
Gyroscopes Bias Stability	≤100.50° per hour
Gyroscopes noise	0.05° per hour
Gyroscopes drift	0.005° per hour
Gyroscopes Nonlinear Degree of Scale Factor	≤20 ppm
Gyroscopes Frequency	50 Hz
Accelerometers Bias Stability	100 µg
Accelerometers Nonlinear Degree of Scale Factor	≤20 ppm
Accelerometers Frequency	50 Hz
GPS Position Error Noise	0.8 m, 0.8 m, 1 m
GPS Velocity Error	0.1 m/s, 0.1 m/s, 0.1m/s
GPS Frequency	1 Hz
Camera FOV	0.3 m
Camera Map Resolution	648 × 488
Camera Frequency	10 Hz

**Table 2:** Estimated position errors with the three integrated methods.

Methods\Errors	Maximum Latitude Error (m)	Maximum Longitude Error (m)	Latitude RMSE (m)	Longitude RMSE (m)	Position RMSE (m)
Method 1 (Pure MEMS–INS)	100.98	110.23	72.543	78.32	106.75
Method 2 (VNBM/GPS/MEMS–INS/Centralized KF)	18.53	19.47	10.43	11.67	15.65
Method 3 (Proposed VNBM/GPS/MEMS using ADSF Combined filter)	0.93	0.82	0.96	0.97	1.53

## Data Availability

Not applicable.
